# Gonad Development and Larvae Distribution of the Manila Clam (*Ruditapes philippinarum*) in the Laizhou Bay Nature Reserve

**DOI:** 10.3390/ani16101507

**Published:** 2026-05-14

**Authors:** Xiang Li, Bin Ma, Jianing Wang, Yu Li, Zengguang Feng, Zengqiang Yin, Lei Chen, Zhongming Huo

**Affiliations:** 1 College of Fisheries and Life Sciences, Dalian Ocean University, Dalian 116023, China; 15141432573@163.com (X.L.); 19841146878@163.com (Y.L.); 13478791300@163.com (Z.F.); 2 Laizhou Mingbo Aquatic Products Co., Ltd., Yantai 261442, China; hereiammabin@163.com; 3 College of Marine Science and Environment, Dalian Ocean University, Dalian 116023, China; 13624162670@163.com (J.W.); zqyin@163.com (Z.Y.); 4 Center for Marine Ranching Engineering Science Research of Liaoning Province, Dalian Ocean University, Dalian 116023, China

**Keywords:** Manila clam, reproductive cycle, larval distribution, benthic population, sediment particle size, Laizhou Bay Nature Reserve

## Abstract

The Laizhou Bay Nature Reserve (Shandong, China) is an important conservation area and seed-producing ground for the Manila clam (*Ruditapes philippinarum*). To clarify its reproductive schedule and recent population status, we examined gonadal development in 2020, monitored meat yield and seawater temperature, surveyed planktonic larvae in 2019, and conducted annual benthic resource surveys from 2019 to 2021 together with sediment particle-size analysis. The observations in 2020 indicated a single spring reproductive cycle. Gonadal development initiated in early April, gonadal maturation predominantly took place throughout April, and spawning reached its peak in May at a water temperature of 16.6–17.8 °C. D-shaped larvae were detected at all sampled stations, indicating widespread larval occurrence. Mean benthic abundance declined from 13,002.2 ind·m^−2^ in 2019 to 8336.7 ind·m^−2^ in 2020 and 5042.4 ind·m^−2^ in 2021, and was weakly but significantly correlated with the 426–850 µm sediment fraction (r = 0.244, *p* < 0.05).

## Simple Summary

The Manila clam (*Ruditapes philippinarum*) is an important germplasm resource in the Laizhou Bay Nature Reserve, China. However, it remains unclear whether the reserve continues to support stable population renewal. We combined histological observations of gonadal development, monthly tracking of meat yield and seawater temperature, surveys of planktonic D-shaped larvae, annual benthic resource assessments, and sediment analysis to clarify the reproductive schedule and current population status of the species. During the 2020 observation period, Manila clams in the reserve showed a single spring reproductive cycle, with gonadal development beginning in early April and spawning peaking in May. Planktonic larvae were widely distributed throughout the reserve, indicating widespread larval occurrence during the survey period. In contrast, benthic resources declined continuously from 2019 to 2021, and the proportion of small individuals decreased over time. Benthic abundance showed only a weak but significant positive association with the 426–850 µm sediment fraction, suggesting that suitable sediment alone cannot explain population maintenance. Although the datasets were collected over different years, direct links between larval occurrence and recruitment should be considered in light of the sampling design. Nevertheless, these findings provide useful baseline data on reproduction, larval occurrence, benthic population status, and habitat characteristics, and offer scientific support for Manila clam resource conservation and habitat management in the reserve.

## 1. Introduction

Filter-feeding bivalves provide important ecosystem services, including the improvement of water clarity, nutrient recycling, and the support of coastal food webs [1]. The Manila clam (*Ruditapes philippinarum*), a member of the family Veneridae, is one of the most commercially important bivalves in East Asia and remains a major fishery and aquaculture species in China [2,3]. Beyond its economic value, this species is also an ecologically important infaunal suspension feeder and can influence soft-bottom benthic assemblages and habitat functioning [4].

Like many marine benthic invertebrates, the life cycle of the Manila clam includes a planktonic larval phase followed by benthic juvenile and adult stages. Population persistence therefore depends not only on adult reproductive output, but also on larval transport, settlement success, and post-settlement survival [5]. In protected seed-producing grounds, these processes may become decoupled: larvae may be broadly present in the water column, whereas benthic replenishment can still fail because of hydrodynamic export, broodstock-related variation in larval quality, environmental stress, habitat connectivity, disturbance, or high mortality after settlement [6,7]. Sediment characteristics are also important because they influence burrowing behavior and habitat suitability in Manila clam [8].

The Laizhou Bay Nature Reserve is an important germplasm conservation area for Manila clams in northern China, and recent work has improved understanding of reproductive dynamics in nearby parts of Laizhou Bay [9]. However, despite its management status, the linkage among reproductive timing, larval occurrence, benthic distribution, and recent resource decline within the reserve remains poorly understood. The objectives of this study were therefore to: (1) describe the annual gonadal cycle and seasonal changes in Manila clam; (2) determine the occurrence and distribution of planktonic larvae; (3) evaluate the changes in benthic clam abundance; (4) evaluate the relationship between benthic clam abundance and sediment particle size.

## 2. Materials and Methods

This study integrated multiple datasets collected over different years to characterize the reproductive schedule, larval occurrence, benthic population status, and habitat conditions of Manila clams in the Laizhou Bay Nature Reserve. To help clarify the schedule of the study, the sampling design is summarized as follows: in 2019, surveys were conducted for water quality, planktonic D-shaped larvae, benthic resources, and sediment particle size; in 2020, gonadal development, meat yield, clam size, and benthic resources were investigated; and in 2021, benthic resources and size composition were further monitored. Because these datasets were collected over different years, the study provides an integrated, temporally structured assessment of reproductive schedule, larval occurrence, benthic resources, and habitat conditions.

### 2.1. Survey Station Setup

**Figure 1:**
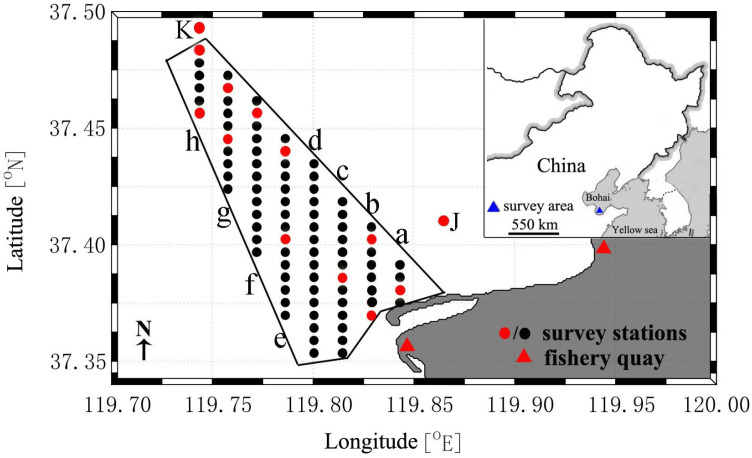
Survey transect and station locations, Laizhou Bay Nature Reserve. Note: Letters a–h indicate the eight survey transects within the reserve, and J and K indicate the two reference stations outside the reserve. Red circles indicate water-quality sampling stations.

The Laizhou Bay Nature Reserve is a subtidal protected area. Eight transects (a–h) were established, including 85 survey stations inside the reserve and 2 reference stations outside the reserve (Figure 1). The same transect framework was used throughout the monitoring program, although the number of stations successfully sampled in a given year varied slightly because some shallow stations could not always be reached safely under field conditions. Survey coordinates were recorded in the field using the Global Positioning System (GPS), and no separate GPS software version was involved.

### 2.2. Observation of Gonadal Development in Adult Clams

From January to December 2020, 50 adult clam specimens were collected monthly from stations a2 and a3 within the reserve. Biological parameters, including shell length, shell width, shell height, total body weight, soft-tissue weight, and gonad weight, were measured. To accurately determine gonadal developmental stages, histological analyses were performed. Gonadal tissue was excised, fixed in Bouin’s solution, dehydrated through a graded ethanol series, cleared with xylene, and embedded in paraffin. Sections were cut at 6 µm and stained with hematoxylin and eosin. The sections were observed and photographed under a trinocular light microscope (XSP-44X.3, Shanghai Optical Instrument Factory, Shanghai, China). The gonadal stage for each month was assigned according to the developmental stage observed in more than 50% of the sections collected that month.

### 2.3. Meat Yield and Seawater Temperature Monitoring

To determine the meat yield (MY), clam samples were collected monthly at the fixed sampling stations from January to December 2020. Meat yield was calculated using the following formula:

MY = (W_f_/W) × 100%

where W_f_ is the weight of the soft tissue, and W is the wet weight of the clam.

To describe the seasonal correspondence between seawater temperature fluctuations and the clam reproductive cycle, concurrent monthly monitoring of seawater temperature was conducted at the aforementioned sampling stations during the same period (January–December 2020). Temperature measurements were performed in the early, middle, and late periods of each month using a YSI 6600 Multiparameter Water Quality Sonde (YSI Inc., Yellow Springs, OH, USA).

### 2.4. Water Quality Sampling and Analysis

On 24 May and 20 July 2019, 11 stations inside and 2 stations outside the reserve (red circles in Figure 1) were sampled during high tide from the research vessel Mingbo (No. Lulaiyang 67771) to investigate water-quality conditions. Water samples (500 mL) were collected at a depth of 1.0 m using a plexiglass water sampler. A YSI 6600 Multiparameter Water Quality Sonde (YSI Inc., Yellow Springs, OH, USA) was used to measure temperature, salinity, dissolved oxygen, pH, total dissolved solids, and water depth in situ. Transparency was measured using a Secchi disc. Nutrient concentrations and chlorophyll a in the water samples were determined according to the marine monitoring specifications [10,11]. Seawater quality grades were assigned according to the National Seawater Quality Standards [12], which specify water-quality requirements for different functional sea areas under China’s jurisdiction.

### 2.5. Collection of D-Shaped Larvae

D-shaped larvae were collected by vertically hauling a Shallow-water III plankton net (mesh size: 77 µm; custom-made according to standard specifications, Dalian, China) at a speed of 0.5 m·s^−1^ from 1 m above the seabed to the surface. Samples were fixed in 95% ethanol. After fixation and concentration, D-shaped clam larvae were identified and counted under a CX41-RF biological microscope (Olympus, Tokyo, Japan).

### 2.6. Molecular Identification Procedure for D-Shaped Larvae

Total DNA was extracted from D-shaped larvae using a TIANamp Marine Animals DNA Kit (DP324, Tiangen Biochemical Technology Co., Ltd., Beijing, China) following the manufacturer’s instructions. Primers specific for amplification of the mitochondrial 16S rRNA gene of Manila clams were synthesized by BGI (Beijing Genomics Institute, Shenzhen, China). The forward and reverse primer sequences were 5′-CGCCTGTTTAHYAAAAACAT-3′ (16S rRNA-F) and 5′-CCGGTCTGAACTCAGMTCAYG-3′ (16S rRNA-R), respectively [13]. PCR amplification and purification of PCR products were performed as described previously [14], with an annealing temperature of 56 °C. Purified PCR products were sequenced using an ABI 3730xl DNA Analyzer (BGI Gene, Shenzhen, China). The resulting sequences were compared against the NCBI GenBank database using BLASTn version 2.16.0+ (National Center for Biotechnology Information, Bethesda, MD, USA).

### 2.7. Collection of Benthic Clams and Sediment

Benthic clams and surface sediment samples (to a depth of 4.0 cm) were collected by divers using SCUBA and shovels within a 15.0 cm × 15.0 cm sampling frame. Samples were washed through 20–mesh silk gauze (approximately 850 µm mesh), and small individuals were separated from the sediment by flotation and sedimentation. The retained material was examined under a microscope. Additional sediment samples were collected using a 32 cm × 20 cm grab sampler (Qingdao Juchuang Shidai Environmental Protection Technology Co., Ltd., Qingdao, China), dried to constant weight at 60 °C, and weighed on an analytical balance (AR224CN, OHAUS Corporation, Parsippany, NJ, USA), and passed through a series of test sieves (5–100 mesh; particle-size range: 150–4000 µm) for particle-size analysis. To determine size-class composition, clams were measured at 10 stations with high abundance (b5, c11, e5, e6, e15, f5, f10, f11, g7, and h1).

### 2.8. Data Analysis and Processing

All data were presented as means ± standard deviations. To objectively assess interannual changes, clam abundance were also examined using the subset of 69 stations that were sampled consistently across 2019–2021. Matlab 2018b (MathWorks, Natick, MA, USA) was used to map the spatial distribution of clam abundance. IBM SPSS Statistics 19.0 (IBM Corp., Armonk, NY, USA) was used for analysis of variance, Duncan’s multiple range tests, and Pearson correlation analyses.

## 3. Results

### 3.1. Gonadal Developmental Stages of the Manila Clam

Based on tissue morphology, gonadal development in the Manila clam was divided into five stages: proliferation, growth, maturation, release, and resting [15].

**Figure 2:**
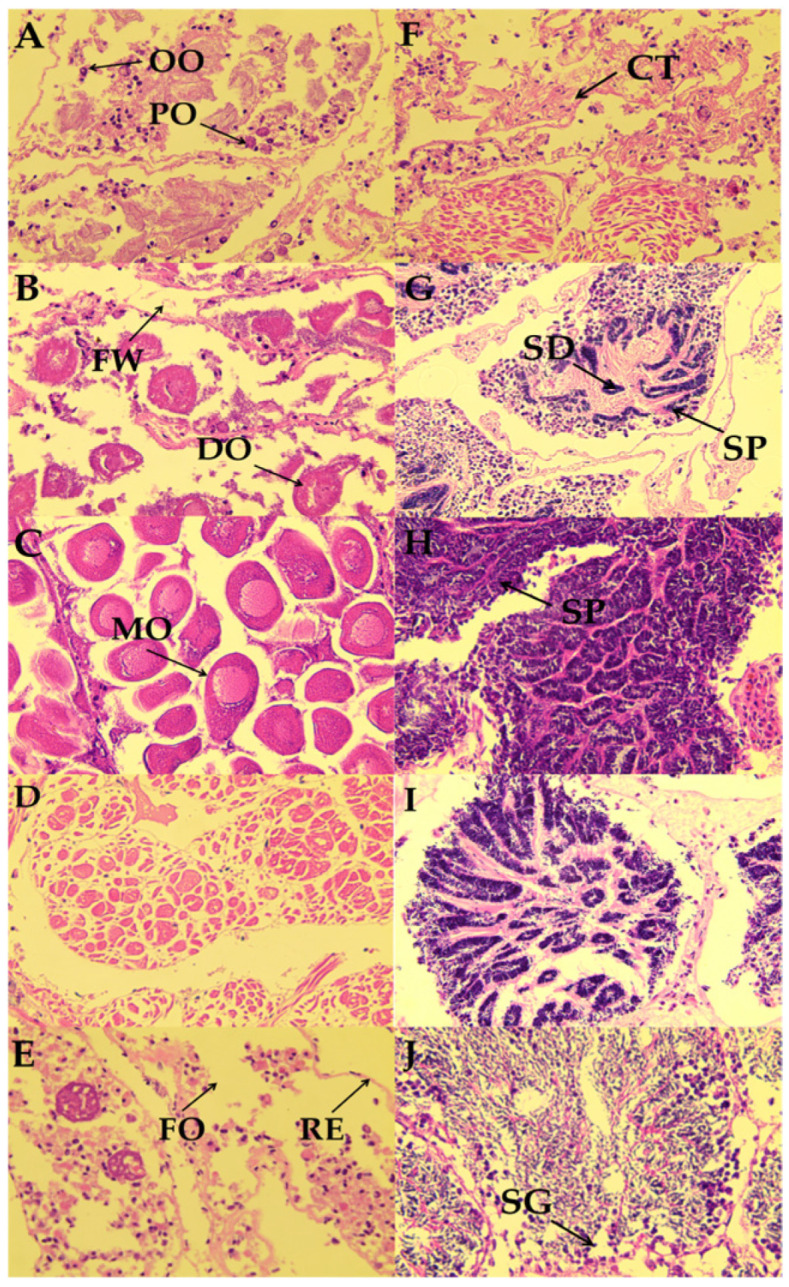
Gonadal developmental stages of the Manila clams in the reserve. Note: (**A**) female proliferation stage (×400); (**B**) female growth stage (×400); (**C**) female maturation stage (×400); (**D**) female release stage (×400); (**E**) female resting stage (×400); (**F**) male proliferation stage (×400); (**G**) male growth stage (×400); (**H**) male maturation stage (×400); (**I**) male release stage (×400); (**J**) male resting stage (×400). OO, oogonia; PO, primary oocyte; FW, follicular wall; DO, degenerated oocyte; MO, mature oocyte; FO, follicle; RE, reproductive epithelium; CT, connective tissue; SD, sperm duct; SP, spermatozoa; SG, spermatogonia. Sections were stained with hematoxylin and eosin (H&E); the purple and pink to reddish colors mainly reflect nuclear and cytoplasmic/connective-tissue staining, respectively.

Ovarian development in the reserve proceeded as follows. During the proliferation stage (early April), the ovaries exhibited a dendritic pattern with few follicles, and primordial germ cells were visible along the follicular walls. During the growth stage (mid-April), irregular to nearly spherical oocytes with prominent nuclei appeared, and yolk granules began to accumulate in the cytoplasm. During the maturation stage (late April to early May), the follicles became fully expanded and were filled with oval oocytes containing distinct yolk granules. During the release stage (mid-May), the oocytes became disorganized, and partial rupture of the follicles formed cavities, indicating batch spawning. During the resting stage (early June), germ cells were largely depleted, the follicular walls had collapsed into cavities, and only scattered degenerating oocytes remained. Representative features of each stage are shown in Figure 2A–E.

Testicular development in the reserve followed a similar sequence. During the proliferation stage (early April), follicles began to develop, spermatogonia were sparse, and the gonads consisted mainly of connective tissue. During the growth stage (mid-April), the number of follicles increased and distinct follicular cavities formed, with spermatogonia arranged in clusters. During the maturation stage (mid- to late April), the follicles became densely packed with spermatozoa. During the release stage (early May), partial sperm release produced patchy empty spaces within the follicles. During the resting stage (mid- to late May through late June), the follicles gradually emptied and atrophied, eventually leaving only connective tissue. Representative features of each stage are shown in Figure 2F–J.

### 3.2. Seasonal Variation in Meat Yield and Seawater Temperature

**Figure 3:**
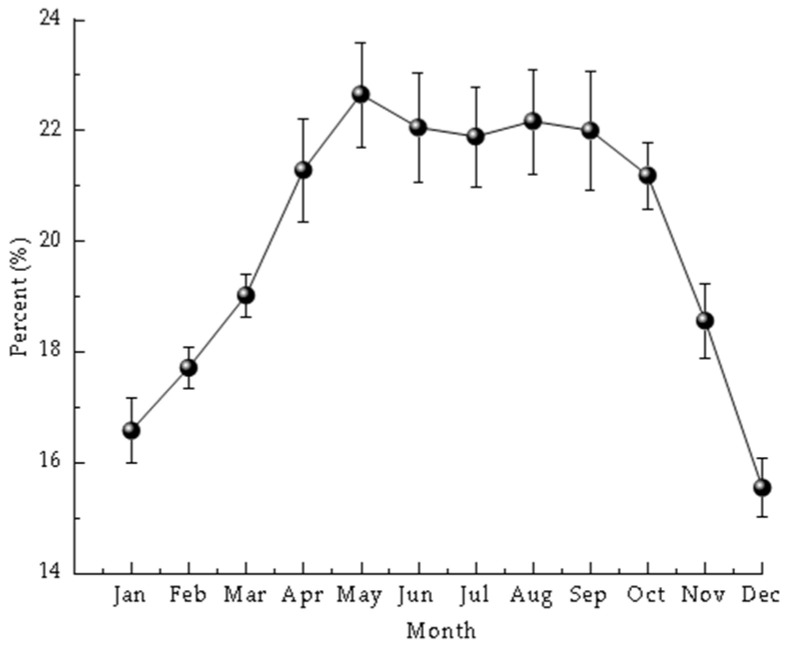
Temporal variation in meat yield of Manila clams in the reserve in 2020.

Annual variation in meat yield is shown in Figure 3. Meat yield displayed an overall bell-shaped seasonal pattern, reaching a maximum in May, which coincided with the main reproductive period and extensive gonadal maturation. After May, meat yield declined sharply, consistent with tissue loss associated with spawning and post-spawning recovery.

**Figure 4:**
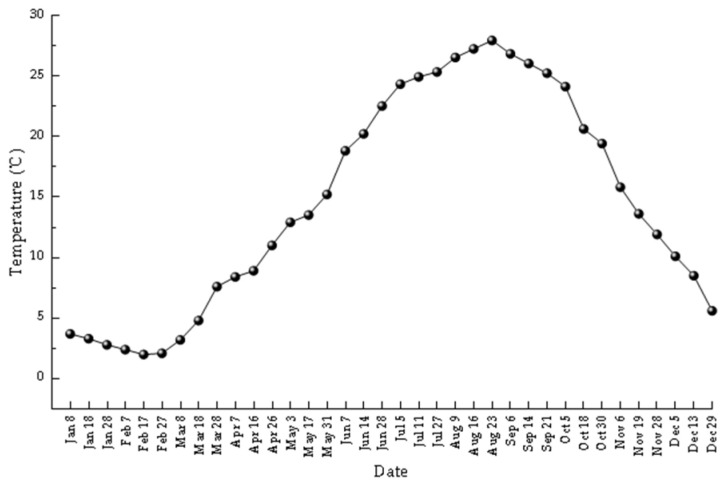
Temporal variation in seawater temperature in the reserve in 2020.

Seawater temperature data are shown in Figure 4. The seasonal temperature curve and the meat yield curve showed broadly similar trends, although the annual temperature maximum occurred later, in late August. During the key transition from gonadal development to spawning (March–May), temperature rose rapidly, which was consistent with a seasonal correspondence between warming and reproductive progression in the reserve.

### 3.3. Size and Individual Weight of the Manila Clam

**Figure 5:**
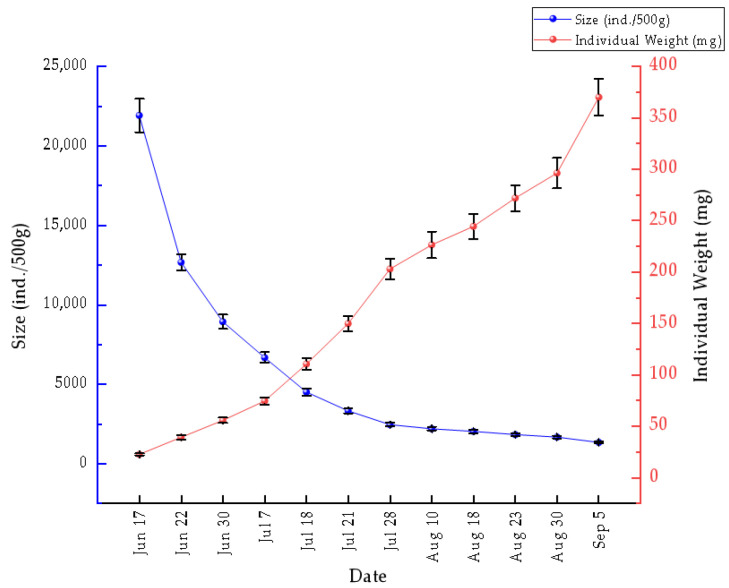
Temporal changes in the individual size of Manila clams in the reserve from June to September 2020.

Changes in size structure and individual weight from June to September 2020 are shown in Figure 5. Abundance, expressed as individuals per unit area, decreased from 21,911.17 ± 1052.22 ind·m^−2^ to 1351.67 ± 68.21 ind·m^−2^, whereas mean individual weight increased significantly from 22.82 ± 0.96 mg to 369.91 ± 17.99 mg over the same period. The most pronounced decrease in abundance and increase in individual weight occurred from mid-June to late July, after which both trends continued at a slower rate during August and September.

### 3.4. Water Quality Conditions Inside and Outside the Reserve

Water quality conditions inside and outside the reserve were compared on 24 May and 20 July 2019 (Table 1). According to the National Seawater Quality Standards [12], dissolved oxygen, pH, and reactive phosphate met Grade I standards at all sampling sites, whereas inorganic nitrogen ranged between Grades I and II. No significant differences in water quality were detected between sites inside and outside the reserve. Nutrient concentrations were slightly higher in nearshore than in offshore waters, particularly in July, although the differences were not significant (*p* > 0.05). Compared with May, seawater transparency decreased in July, whereas nutrient and chlorophyll–*a* concentrations increased. Reactive phosphate and chlorophyll–*a* showed significant differences between the two sampling periods (*p* < 0.05).

### 3.5. Molecular Identification of Larvae

The purified PCR products were 527 bp in length. BLAST analysis showed that, at 97.0% query coverage, the sequences shared more than 99.6% identity with the mitochondrial 16S rRNA sequence of *R. philippinarum* isolate TaRph (Sequence ID: JN969951.1), indicating that the collected larvae were Manila clam larvae.

### 3.6. Distribution of Planktonic Larvae in the Reserve

**Figure 6:**
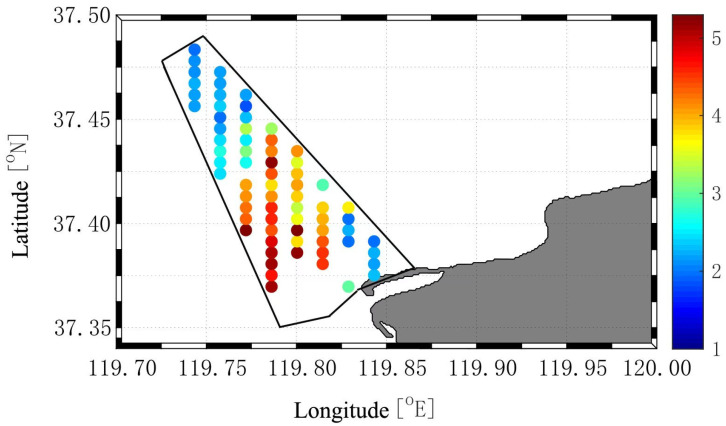
Spatial distribution of planktonic D-shaped Manila clam larvae in the reserve in 2019. Note: Data are expressed as log_10_X, where X is the abundance of planktonic larvae (ind·m^−3^).

Of the 85 planned survey stations within the reserve, 69 were successfully sampled, whereas the remaining 16 could not be surveyed because the water was too shallow for safe operation of the research vessel. D-shaped Manila clam larvae were detected at all sampled stations, with abundances ranging from 67.1 to 304,580.2 ind·m^−3^ and an overall mean of 25,849.5 ind·m^−3^. Larval abundances were 1–100 ind·m^−3^ at 6 stations (8.69%), 101–1000 ind·m^−3^ at 24 stations (34.78%), 1001–10,000 ind·m^−3^ at 13 stations (18.84%), 10,001–100,000 ind·m^−3^ at 19 stations (27.53%), and >100,000 ind·m^−3^ at 7 stations (10.14%) (Figure 6). The highest larval abundances were observed along the central transects of the reserve.

### 3.7. Distribution of Benthic Clams in the Reserve

**Figure 7:**
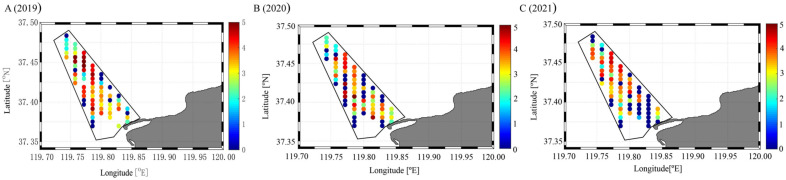
Spatial distribution of benthic Manila clams in the reserve in (**A**) 2019, (**B**) 2020, and (**C**) 2021. Note: Data are expressed as log_10_X, where X is the abundance of benthic Manila clams (ind·m^−2^).

In 2019, the abundance of benthic clams at the 69 surveyed stations in the reserve ranged from 0 to 126,520.7 ind·m^−2^, with a mean of 13,002.2 ind·m^−2^. The abundance was 0 ind·m^−2^ at 12 stations (17.39%), 1–100 ind·m^−2^ at 6 stations (8.69%), 101–1000 ind·m^−2^ at 11 stations (15.94%), 1001–10,000 ind·m^−2^ at 19 stations (27.53%), 10,001–100,000 ind·m^−2^ at 19 stations (27.53%), and >100,000 ind·m^−2^ at 2 stations (2.90%). Of the 21 stations with densities exceeding 10,000 ind·m^−2^, 19 were located along transects e, f, and g, primarily in the middle and offshore sections of these transects (Figure 7A).

In 2020, the abundance of benthic clams at the 74 surveyed stations in the reserve ranged from 0 to 121,899.0 ind·m^−2^, with a mean of 8336.7 ind·m^−2^. The abundance was 0 ind·m^−2^ at 23 stations (31.08%), 1–100 ind·m^−2^ at 4 stations (5.41%), 101–1000 ind·m^−2^ at 12 stations (16.22%), 1001–10,000 ind·m^−2^ at 21 stations (28.38%), 10,001–100,000 ind·m^−2^ at 13 stations (17.57%), and >100,000 ind·m^−2^ at 1 station (1.35%). Stations with relatively high densities were mainly distributed in the middle sections of the reserve, whereas nearshore and offshore areas generally exhibited lower densities (Figure 7B).

In 2021, the abundance of benthic clams at the 74 surveyed stations in the reserve ranged from 0 to 37,893.0 ind·m^−2^, with a mean of 5042.4 ind·m^−2^. The abundance was 0 ind·m^−2^ at 27 stations (36.49%), 1–100 ind·m^−2^ at 4 stations (5.41%), 101–1000 ind·m^−2^ at 7 stations (9.46%), 1001–10,000 ind·m^−2^ at 24 stations (32.43%), and 10,001–100,000 ind·m^−2^ at 12 stations (16.22%). Stations with relatively high clam abundance were mainly distributed in the middle sections of the reserve, whereas nearshore areas generally exhibited lower abundance (Figure 7C).

### 3.8. Size Composition of Benthic Clams

**Figure 8:**
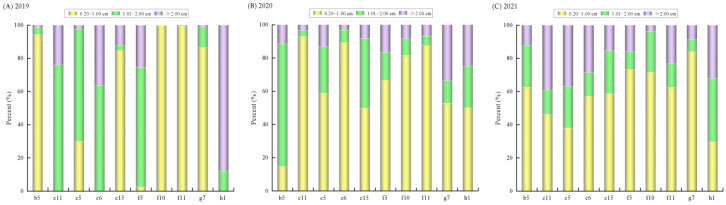
Comparison of size composition of benthic Manila clams at high-abundance stations in the reserve from 2019 to 2021.

Monitoring from 2019 to 2021 showed that the core distribution area of benthic clams remained concentrated in the middle region of the reserve. The proportion of small individuals (shell length 0.2–1.0 cm) decreased progressively, from 77.83% in 2019 to 70.88% in 2020 and 58.75% in 2021, whereas the proportion of large individuals (shell length > 2.0 cm) increased from 4.23% to 13.41% and 21.29% over the same period (Figure 8). The relatively stable sedimentary environment in the middle area, characterized by a high proportion of 426–850 µm sand, may provide favorable habitat conditions for the persistence of this population.

### 3.9. Correlation Between Benthic Clam Abundance and Sediment Particle Size

**Figure 9:**
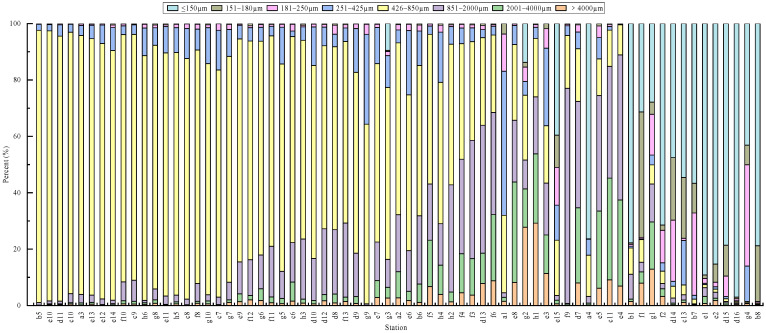
Composition of sediment particle size in the reserve in 2019. Note: Stations were ordered from high (left) to low (right) according to the percentage contribution of the 426–850 µm sediment fraction to the total sample.

In 2019, benthic clam abundance exceeded 1000 ind·m^−2^ at 40 stations. Of these, 29 stations were dominated by sandy sediments in the 426–850 µm size fraction, with contents > 50.13%. Among the remaining 11 stations, 9 were dominated by sandy sediments in the 426–2000 µm fraction, with contents > 40.93%. However, e15 and g1, both located at the edge of the reserve, were notable for their relatively high proportions of muddy sediments ≤ 150 µm, accounting for 39.57% and 27.88%, respectively. Pearson correlation analysis showed that the correlation coefficients (r) between clam abundance and sediment particle-size fractions ranged from 0.027 to 0.244. A significant positive correlation was observed between clam abundance and the proportion of 426–850 µm sediment (r = 0.244, *p* < 0.05; Table 2 and Figure 9).

## 4. Discussion

### 4.1. Gonadal Developmental Pattern of the Manila Clam in the Reserve

In shellfish reproductive biology, gonadal development has long been an important focus of research. Manila clams are dioecious, but they lack obvious external secondary sexual characteristics, making visual sex identification difficult [16]. Previous studies have mainly addressed their reproductive cycle, larval morphology, and early post-settlement development [17]. In this study, based on observations conducted in 2020, spring appeared to be the main reproductive season for Manila clams in the reserve. Peak gamete release occurred in mid-May, and ovarian development followed the sequence of proliferation, growth, maturation, release, and resting, with clear cytological features at each stage. This developmental pattern is generally consistent with that reported for other bivalves, such as *Meretrix meretrix* and *Cerastoderma edule* [18,19], suggesting that the core process of germ-cell development is evolutionarily conserved. However, marked differences in reproductive timing are evident among species. Manila clams in the reserve exhibited a single annual reproductive cycle during the 2020 observation period, whereas *M. meretrix* exhibits a tropical double cycle and *P. undulatus* has a longer subtropical reproductive season. These differences most likely reflect species-specific adaptations to local environmental conditions. Among the environmental factors discussed in previous studies, seawater temperature is often considered important because it can influence germ-cell development and energy accumulation, thereby influencing the duration and frequency of reproductive cycles.

Field monitoring showed that reproductive development and spawning occurred during a period of seasonal temperature increase. During the critical reproductive period, from gonadal development to gamete release (approximately March to May), seawater temperature exhibited a rapid increase, from roughly 8.5 °C in the proliferation stage to 13.5 °C during the release stage. During embryonic and larval development, water temperature remained relatively high, with an annual mean of 15.6 °C. These observations indicate that the reproductive period of the Manila clam coincides with the seasonal rise in seawater temperature. Although this study did not formally test the statistical relationship between temperature and gonadal development, the observed seasonal correspondence suggests that spring warming may be associated with reproductive development in the reserve. In addition, monitoring of shell-length and body weight in summer 2020 suggested a marked post-spawning shift in energy allocation. After the major release of gametes in mid-May, mean individual weight increased 16.21-fold from June to September, while clam abundance declined over the same period. This pattern likely reflects a reallocation of energy toward somatic growth, tissue recovery, and reserve accumulation after spawning. More broadly, reproductive cycles in bivalves may be regulated not only by temperature but also by food availability and broader seasonal environmental conditions, which together influence growth and gametogenesis [20,21].

In tropical waters, characterized by persistently warm conditions and a more stable food supply, bivalves are often associated with double reproductive cycles or prolonged spawning seasons, whereas distinct seasonality in temperate waters tends to favor a compact single annual cycle. In addition to temperature, salinity may also play an important regulatory role in Manila clam reproduction. Previous studies have shown that salinity shifts can affect osmotic balance in bivalves and may thereby influence spawning behavior, gamete viability, and the success of early embryonic development [22]. Furthermore, Manila clams have a relatively short life cycle. After fertilization and hatching, larvae remain planktonic for nearly 20 days before settling into the benthic habitat. This life-history trait provides a clear temporal window for subsequent field investigations of planktonic larvae.

### 4.2. Water Quality in the Reserve

Laizhou Bay is relatively shallow, and water temperature rises rapidly in spring. The reserve is located close to shore, with water depths ranging from 3.8 to 13.2 m at high tide and mostly between 6.0 and 8.0 m. Such conditions may be favorable for gonadal development and spawning in Manila clams. In this study, no significant differences in seawater quality were detected between sites inside and outside the reserve during the two sampling occasions in 2019, which may reflect the vigorous water exchange dynamics across the study area at the time of observation [23]. Since water quality was only surveyed on two sampling occasions, these findings should be regarded as short-term snapshots. Planktonic larvae of Manila clams are sensitive to salinity. When surface salinity decreases after heavy rainfall, larval distribution may shift toward areas with salinity around 30 [24]. In 2019, no substantial rainfall occurred along the Bohai Sea coast during the survey period, and salinity inside the reserve did not decrease markedly at the time of sampling.

The southeastern part of the reserve, adjacent to the Sanshan Island fishery wharf, is bordered by residential areas and aquatic-product processing facilities. Human activities and land-based runoff may therefore influence nutrient concentrations in nearby coastal waters, especially in nearshore areas during summer. From May to July, water temperature increases rapidly in this relatively shallow reserve, and nutrient-rich nearshore waters may promote phytoplankton growth, thereby providing food resources for clams. Nevertheless, longer-term environmental monitoring would further clarify how seasonal water-quality variation influences population dynamics in the reserve.

### 4.3. Distribution of Planktonic Larvae and Benthic Clams

D-shaped larvae were detected at all 69 surveyed stations in the reserve in 2019, indicating a widespread spatial distribution. Larval distribution is influenced by seawater movement driven by tides and wind [25,26], especially when larval horizontal swimming ability is lower than current speed or when vertical swimming speed is similar to the vertical velocity of surrounding water masses [27]. However, the southwest–northeast current regime around the reserve [28] is nearly perpendicular to the nearshore–offshore orientation of the reserve, suggesting that tidal transport may have limited influence on the cross-shore distribution of larvae. Based on this pattern, larval distribution within the reserve may therefore be more closely related to the distribution of adult spawning stocks. Laboratory studies have shown that at 20 °C, Manila clams can develop into D-shaped larvae of approximately 100 µm within two days [29], remain planktonic for about two weeks, and then metamorphose and settle at shell lengths of 200–300 µm [30]. Larvae measured in this study ranged from 100 to 200 µm, which may reflect asynchronous gonadal development and spawning within the adult population.

Monitoring from 2019 to 2021 further revealed a decline in benthic clam resources within the reserve. Although annual station coverage was not completely identical because some sites were inaccessible during field surveys, the same declining pattern was retained when the analysis was restricted to the 69 stations sampled consistently across all three years, supporting the interpretation that the temporal trend was not solely driven by differences in station coverage. Across all surveyed stations, mean abundance decreased from 13,002.2 ind·m^−2^ to 5042.4 ind·m^−2^, accompanied by a contraction in the distribution range, a reduction in the proportion of high-abundance stations, and an increase in zero-abundance stations. These trends suggest a weakening of the benthic population over time. The underlying drivers remain to be further clarified. Possible explanations may include fishing pressure, habitat alteration, and increasing coastal disturbance, all of which have been reported as important influences in other nearshore marine ecosystems [31]. However, because these factors were not directly quantified in this study, they should be regarded as hypotheses rather than confirmed causes of the observed decline. Future studies should focus on quantifying the influence of these factors.

Despite the wide distribution of planktonic larvae, no significant correlation was found between larval abundance and benthic abundance. This may reflect the influence of environmental factors such as temperature, salinity, and hydrodynamic conditions on larval settlement [32], as well as variation in post-settlement survival and growth that ultimately shape benthic population abundance [33]. Because the reproductive, larval, benthic, and environmental datasets were obtained in different sampling periods rather than through a fully synchronized monitoring design, direct links between larval occurrence and subsequent benthic recruitment should be interpreted cautiously under the present sampling design. At the same time, shell-length composition changed markedly from 2019 to 2021. The proportion of small individuals declined from 77.83% in 2019 to 58.75% in 2021, whereas the proportion of large individuals increased from 4.23% to 21.29%. This shift indicates that, under declining overall abundance, the population structure gradually became dominated by larger clams. This pattern may be consistent with reduced juvenile replenishment while surviving individuals continued to grow into larger size classes. Because settlement success, juvenile survival, and post-settlement mortality were not directly monitored, this interpretation should be viewed as a plausible explanation that warrants further testing. Although water quality in the reserve met the relevant standards during the survey period, the observed decline in benthic abundance over the monitoring period warrants attention. From a management perspective, long-term water quality monitoring and habitat maintenance may be beneficial for the conservation of Manila clam resources in the reserve. Potential measures include restricting harvesting during the reproductive peak in May, exploring harvest size limits consistent with the growth characteristics of the species, and protecting key habitats to reduce human disturbance.

### 4.4. Relationship Between Benthic Distribution and Sediment Particle Size

Infaunal bivalves burrow into sediment to avoid predation and physical stress such as desiccation [34]. Accordingly, benthic organisms often show strong associations with the particle-size characteristics of the sediments they inhabit [35,36]. Many species are adapted to medium or fine sediments, including *Donax trunculus* [37], *Donax variabilis* [38], and *Macoma balthica* [39]. Sediment uniformity can also strongly influence molluscan distribution [40].

Sandy sediments are generally considered most suitable for Manila clam survival [41]. Manila clams are usually most common in habitats with 70–80% sand and occur less frequently in muddy flats with low sand content [42]. Previous studies have shown that the burrowing rate of adult Manila clams does not differ significantly among coarse (500–2000 µm), medium (250–500 µm), fine (62–250 µm), and silty (<62 µm) sands [43]. In this study, benthic abundance was significantly and positively correlated with the proportion of 426–850 µm sediment (*p* < 0.05). Although the correlation was relatively weak (r = 0.244), it indicates that the 426–850 µm sediment fraction may be associated with favorable benthic habitat conditions. Therefore, this sediment fraction should be considered as one habitat-related factor associated with benthic distribution, rather than as the sole controlling factor. One possible explanation is that the population sampled here was dominated by relatively small individuals (shell length 0.2–1.0 cm), whose burrowing performance and habitat requirements may differ from those of larger clams [44]. Burrowing in bivalves depends on coordinated shell movement and pressure generated by the foot and hemocoel [45]. In mud and very fine sands, sediment compactness may increase resistance to burrowing, especially for newly settled juveniles. By contrast, if sediment particles are too coarse, newly settled larvae may require more energy to burrow completely into the substrate. Thus, an intermediate sandy substrate may be associated with relatively favorable conditions in this system. Nevertheless, given the weak correlation observed, sediment characteristics should be considered together with other potentially important factors, including local environmental conditions, post-settlement survival, habitat quality, and human disturbance. At stations e15 and g1 on the edge of the reserve, however, sediments were relatively muddy (39.6% and 27.9%, respectively), yet clam abundance remained relatively high. At neighboring stations (e.g., e13, e14, f10, and f11), clams were highly abundant. This pattern further suggests that sediment alone cannot fully explain local benthic abundance. One possible interpretation is that local abundance patterns may also be influenced by nearby favorable habitats or other unmeasured environmental factors. Further field observations would help clarify the contribution of these factors to local abundance patterns.

## 5. Conclusions

This study showed that, during the 2020 observation period, gonadal development of the Manila clam in the Laizhou Bay Nature Reserve followed a single reproductive cycle in spring, with a spawning peak in May. Planktonic larvae were widely distributed across the reserve, whereas benthic abundance declined from 2019 to 2021, accompanied by a contraction in the observed distribution range. No significant correlation was detected between larval abundance and benthic abundance in the present dataset. However, because larval, benthic, reproductive, and environmental data were collected in different years, the observed relationship among larval occurrence, recruitment, and benthic population changes should be interpreted with appropriate caution. In addition, benthic abundance was weakly but significantly positively correlated with the proportion of 426–850 µm sandy sediment (*p* < 0.05), suggesting that sediment characteristics may be one of several factors associated with benthic distribution, but are unlikely to fully explain the observed variation on their own. Overall, these findings provide baseline information on reproduction, larval occurrence, water quality, benthic resources, and sediment characteristics of Manila clams in the reserve, thereby offering a scientific basis for resource management and ecological conservation. Future work should prioritize concurrent monitoring of larval abundance, settlement, recruitment, and environmental conditions to better evaluate natural replenishment processes of Manila clam populations. From a precautionary management perspective, protection during the reproductive period, habitat maintenance, and appropriately regulated harvesting may help support the long-term conservation of Manila clam germplasm resources in the reserve.

## Figures and Tables

**Table 1:** Seawater quality conditions inside and outside the reserve.

	24 May 2019		20 July 2019	
	Inside the Reserve	Outside the Reserve	Inside the Reserve	Outside the Reserve
Water temperature/°C	19.2–20.5/20.1 ^a^	19.8–20.7/20.3 ^a^	24.4–25.2/25.0 ^b^	24.8–25.7/25.3 ^b^
Salinity	25.4–25.9/25.6 ^a^	25.8–25.9/25.9 ^a^	26.5–27.0/26.8 ^a^	26.2–27.1/26.7 ^a^
pH	8.17–8.24/8.22 ^a^	8.18–8.21/8.20 ^a^	8.22–8.29/8.26 ^a^	8.24–8.27/8.26 ^a^
Dissolved oxygen/mg·L^−1^	6.90–7.44/7.05 ^a^	6.92–7.14/7.03 ^a^	6.55–7.82/7.16 ^a^	6.61–7.78/7.20 ^a^
Total dissolved solids/mg·L^−1^	25.4–25.9/25.8 ^a^	25.8–25.9/25.9 ^a^	28.7–30.2/29.1 ^b^	28.2–30.7/29.5 ^b^
Transparency/m	3.5–5.0/4.3 ^a^	3.7–4.5/4.1 ^a^	3.1–4.3/3.8 ^a^	3.3–4.1/3.7 ^a^
Water depth/m	3.8–13.0/9.6 ^a^	8.1–12.5/10.3 ^a^	4.0–13.2/9.7 ^a^	8.2–12.8/10.5 ^a^
NO_2_–N/mg·L^−1^	0.002–0.003/0.002 ^a^	0.002–0.002/0.002 ^a^	0.002–0.006/0.004 ^a^	0.002–0.004/0.003 ^a^
NO_3_–N/mg·L^−1^	0.06–0.10/0.08 ^a^	0.07–0.08/0.08 ^a^	0.09–0.12/0.10 ^ab^	0.10–0.12/0.11 ^b^
NH_3_–N/mg·L^−1^	0.08–0.11/0.10 ^a^	0.08–0.09/0.09 ^a^	0.10–0.15/0.13 ^b^	0.11–0.13/0.12 ^b^
Inorganic nitrogen/mg·L^−1^	0.14–0.21/0.20 ^a^	0.16–0.21/0.19 ^a^	0.19–0.28/0.23 ^a^	0.19–0.22/0.21 ^a^
Active phosphate/mg·L^−1^	0.003–0.006/0.004 ^a^	0.004–0.005/0.005 ^a^	0.004–0.011/0.009 ^b^	0.006–0.010/0.008 ^b^
Chlorophyll *a*/µg·L^−1^	1.05–3.94/2.49 ^a^	1.25–3.88/2.57 ^a^	1.31–4.32/3.01 ^b^	1.53–4.24/2.89 ^b^

Note: Data are presented as range/mean values. Different superscript letters within the same row indicate significant differences (*p* < 0.05).

**Table 2:** Pearson correlation coefficients between benthic clam abundance and sediment particle size.

	Sediment Types and Sediment Particle Size/µm							
	Mud	Sand						
	≤150	151–180	181–250	251–425	426–850	851–2000	2001–4000	>4000
Pearson correlation	−0.236	−0.151	−0.142	−0.107	0.244 *	0.136	−0.027	−0.090
Sig. (2-tailed)	0.050	0.215	0.246	0.383	0.043	0.266	0.823	0.463

Note: * indicates that the correlation is significant at the 0.05 level (2–tailed).

## Data Availability

Data will be made available on request.
